# An Unusual Congenital Heart Disease: Giant Left Atrial Appendage

**DOI:** 10.14797/mdcvj.1059

**Published:** 2022-03-14

**Authors:** Julio C. Sauza-Sosa, Erika L. De la Cruz-Reyna, Carlos N. Velazquez-Gutierrez

**Affiliations:** 1 Centro Hospitalario MAC Periferico Sur, Mexico City, Mexico

**Keywords:** congenital heart disease, left atrial appendage

## Abstract

A 52-year-old female was admitted to our hospital in April 2021 with dyspnea. She was discharged from the hospital 3 weeks ago due to the diagnosis of pneumonia caused by coronavirus disease 2019 (COVID-19). Physical examination revealed an oxygen desaturation of 82%. The patient underwent computed tomography angiography (CTA) that showed a ground-glass pattern and a giant left atrial appendage (***Figure 1A***). Film array respiratory panel was negative, and pulmonary aspergillosis was diagnosed after bronchoscopy. Cardiac magnetic resonance corroborated the huge left atrial appendage (***Figure 1B***). No other structural or functional heart abnormalities were diagnosed.

A giant left atrial appendage is a rare cardiac anomaly that can be congenital or acquired. In the literature, it is called a left atrial appendage aneurysm. The dilatation can be generalized or focused. Although it can occur in all age groups, it is predominant in patients in their 30s to 50s and most common in females.^1^ Patients can be asymptomatic or present with symptoms such as palpitations, chest pain, or dyspnea. A number of recent cases in the literature have highlighted the diagnostic utility of CTA.^2^ While there is no standard treatment for this condition, surgical resection is the most frequent therapy. Another option reported in the literature is anticoagulant treatment for select cases.^3^ Closure of the left atrial appendage is a more recent and emerging intervention that can be considered.

In our patient, we initiated anticoagulant therapy to reduce the risk of thromboembolic events; however, we recommended left atrial appendage occlusion or surgical resection after completing the treatment for pulmonary aspergillosis.

## Consent for Publication

The corresponding author had a written consent of the patient to use the data for publication.

**Figure 1:**
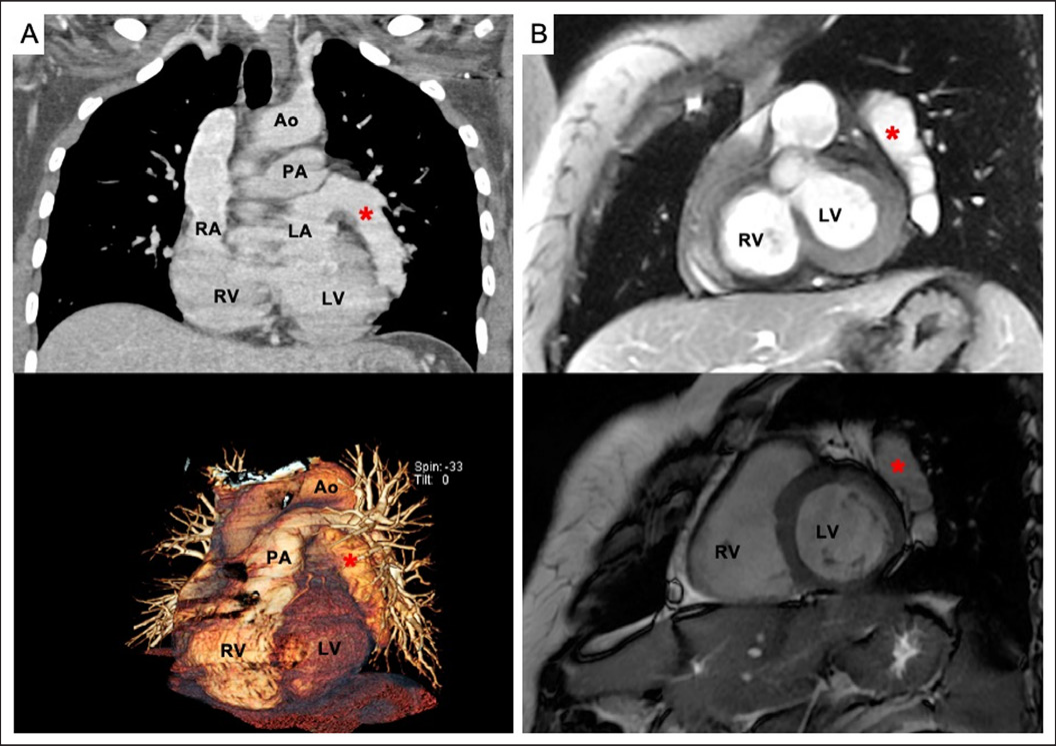
**(A)** Computed tomography angiography showed a ground-glass opacity and a giant left atrial appendage (red asterisk). **(B)** Cardiac magnetic resonance confirmed the huge left atrial appendage (red asterisk). Ao: aorta: PA: pulmonary artery; RA: right atrium; RV: right ventricle; LA: left atrium; LV: left ventricle.
